# Case Report: Renal potassium wasting in SARS-CoV-2 infection

**DOI:** 10.12688/f1000research.24621.2

**Published:** 2020-11-13

**Authors:** Holly Mabillard, Hilary Tedd, Ally Speight, Christopher Duncan, David A. Price, John A. Sayer

**Affiliations:** 1 Renal Services, The Newcastle Hospitals NHS Foundation Trust, Newcastle upon Tyne, Tyne and Wear, NE77DN, UK; 2 Department of Respiratory Medicine, The Newcastle Hospitals NHS Foundation Trust, Newcastle upon Tyne, Tyne and Wear, NE14LP, UK; 3 Department of Gastroenterology, The Newcastle Hospitals NHS Foundation Trust, Newcastle upon Tyne, Tyne and Wear, NE14LP, UK; 4 Department of Infection and Tropical Medicine, The Newcastle Hospitals NHS Foundation Trust, Newcastle upon Tyne, Tyne and Wear, NE14LP, UK; 5 Translational and Clinical Research Institute, Newcastle University, Newcastle upon Tyne, Tyne and Wear, NE13BZ, UK; 6 NIHR Newcastle Biomedical Research Centre, Newcastle University, Newcastle upon Tyne, Tyne and Wear, NE45PL, UK

**Keywords:** COVID-19, hypokalaemia, potassium, tubulopathy

## Abstract

Severe acute respiratory syndrome coronavirus 2 (SARS-CoV-2) infection is associated with many potentially fatal complications. Renal involvement in various forms is common in addition to serum electrolyte disturbances. Early reports suggest that hypokalaemia may frequent those with SARS-CoV-2 infection and various aetiological factors may cause this electrolyte disturbance. A Chinese retrospective study has demonstrated renal potassium wasting in patients infected with SARS-CoV-2, however, it is not known if these patients were receiving diuretic therapy which may be a contributing factor. This case report illustrates an example of renal potassium wasting in SARS-CoV-2 infection in the absence of diuretics and extra-renal mechanisms with important lessons learned.

## Introduction

Severe acute respiratory syndrome coronavirus 2 (SARS-CoV-2) infection is associated with many potentially fatal complications ^1^. Renal involvement in various forms is common in addition to serum electrolyte disturbances ^2^. Renal pathologies identified so far include acute tubular injury, proteinuria, rhabdomyolysis, secondary focal segmental glomerulosclerosis and possible renin-angiotensin-aldosterone system (RAS) activation ^2^.

Early reports suggest that hypokalaemia may frequent those with SARS-CoV-2 infection ^3^ and various aetiological factors may cause this electrolyte disturbance such as gastrointestinal potassium loss, diuretic-induced potassium wasting, renal tubulopathy and RAS activation. An early Chinese retrospective study has demonstrated renal potassium wasting in patients infected with SARS-CoV-2, however it is not known if these patients were receiving diuretic therapy which may have been a contributing factor ^3^. This case report illustrates an example of renal potassium wasting in SARS-CoV-2 infection in the absence of diuretics and extra-renal mechanisms in addition to lessons learned from an important and potentially dangerous complication of the disease. 

## Case report

**Figure 1.**
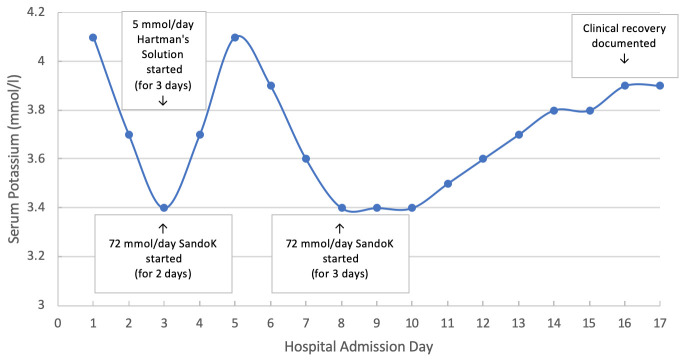
Serum potassium changes during Severe acute respiratory syndrome coronavirus 2 (SARS-CoV-2) infection.

Our patient, an 82 year old Caucasian female, was admitted to the medical admissions unit with confusion and drowsiness in April 2020. She was noted to be pyrexial (38.6 degrees Celsius) and hypoxic (oxygen saturations 86% on room air) on admission and had a Glasgow coma score of 14/15. She had a chronic cough with intermittent clear sputum. Bilateral inspiratory crackles were reported on chest auscultation and she looked hypovolaemic. Blood pressure was 90/60 mmHg and heart rate was 100 bpm. Her comorbidities include chronic obstructive pulmonary disease, previous dynamic hip screw following a right fractured neck of femur, osteoarthritis, hypertension, thoracoabdominal aortic aneurysm and osteoporosis. Usual medications included oral cholecalciferol 1600 units daily, diazepam 2mg nocte as required, lercanidipine 10mg daily, omeprazole 20mg daily, sertraline 50mg daily, tolterodine 1mg twice daily and a salbutamol inhaler. Her chest x-ray did not show evidence of infection. Her diazepam and antihypertensives were suspended and she was commenced on oxygen therapy aiming for saturations between 88 and 92%. Her coronavirus disease 2019 (COVID-19) RT-PCR test from a deep throat swab was positive. The patient developed systemic symptoms the next day and a rise in C-reactive protein (CRP) was seen ( Table 1) so she was commenced on doxycycline 100mg once daily for five days to cover a lower respiratory tract infection. Both urine and blood cultures were negative. 3 Days after admission she developed hypokalaemia which was treated with 72 mmol oral SandoK (potassium bicarbonate and potassium chloride therapy) daily (in divided doses) and she was given 1L intravenous Hartman’s solution (containing 5 mmol/L potassium) daily for three days ( Figure 1). Six days after admission the patient’s oxygen requirements had returned to baseline but hypokalaemia (serum potassium 3.4 mmol/L) persisted despite treatment. A trans-tubular potassium gradient was found to be significantly elevated at 10.7. Her serum potassium level normalised to 3.9 mmol/L eight days later. Clinical improvement was noted at this point and the patient received physiotherapy until her mobility was safe enough to be discharged after a total of 20 days in hospital. Given her COVID-19 positive status she received four weeks of rivaroxaban therapy post discharge to reduce the chance of venous thromboembolism. At no point did our patient have diarrhoea or receive any medications that would interfere with her serum or urine potassium such as RAS inhibitors or loop or thiazide diuretics. Her renal function remained at baseline throughout her admission (serum Creatinine 60–77 µmol/L and eGFR 70–82 mL/min/1.73m ^2^).

**Table 1.** Biochemical tests.

Test	Baseline Results (start of admission)	Patient Results	Normal Values
Blood pH	7.38	7.48	7.35-7.42
pCO2 (kPa)	6.0	6.2	4.5-6.0
Baseline serum potassium (mmol/L)	5.4	4.1	3.5-5.3
Nadir serum potassium (mmol/L)	-	3.4	3.5-5.3
Serum bicarbonate (mmol/L)	25.6	32	21-28
Peak CRP (mg/L)	23	152	<5
Peak Ferritin (µ/L)	237	555	<200 in pre-menopausal women and <300 in men and post-menopausal women
Peak Lactate Dehydrogenase (unit/L)	-	216	135-225
Peak Procalcitonin (ng/mL)	-	0.85	<0.05
TTKG	-	10.7	>4.0 indicates renal potassium loss
Plasma renin (mIU/L)	-	6.7	<59.7
Plasma Aldosterone (pmol/L)	-	<103	103-859
Aldosterone/Renin Ratio (pmol/mIU)	-	<16	<30
Urine creatinine (mmol/l)	-	7.9	-
Urine sodium (mmol/l)	-	7.9	-
Urine potassium (mmol/l)	-	75	-
Urine osmolality (mOsm/Kg)	-	565	-

## Discussion

The renal complications that are known from this devastating disease so far include proteinuria, acute tubular injury, rhabdomyolysis, secondary focal segmental glomerulosclerosis and possible RAS activation (both directly and indirectly) ^2^. This case demonstrates evidence of renal potassium wasting as an example of a further renal complication of SARS-CoV-2 infection which has, so far, only been reported in China yet without the knowledge of contribution from diuretics. Although, the patient’s chronic obstructive pulmonary disease would naturally lead us to speculate this as a cause for her metabolic alkalosis; her serum bicarbonate has always been normal, her COPD was mild, she had an elevated blood pH despite high pCO2 levels and renal potassium wasting is not a known sequela of COPD. Renal potassium wasting was transient and the patient had never before suffered with hypokalaemia, been treated with diuretics, laxatives, mineralocorticoid agonists or had diarrhoea. This would exclude chronic causes of renal potassium wasting such as Glucocorticoid Remedial Aldosteronism, adrenal adenoma/carcinoma, Bartter’s syndrome, Gitelman Syndrome and other disorders of steroid metabolism. She did not have a history of liquorice ingestion or exogenous steroid use. She was not Cushingoid or hypertensive and, when measured, she did not have hyperreninaemia or hyperaldosteronism. It is therefore likely that she had direct tubular loss of potassium probably secondary to direct tubular injury. This could have been a result of her documented hypotension causing acute tubular necrosis but she did not have acute kidney injury or any significant eGFR drop during admission which you would expect to justify her hypokalaemia. Our hypothesis of direct viral injury of the tubular epithelium however, could explain this tubular loss of potassium in the absence of an eGFR decline although this cannot be proven in this patient.

A retrospective pre-print Chinese study highlighted hypokalaemia as a complication early on in the pandemic which contributed to the momentum in speculation of RAS involvement ^3^. This was due to chronic kidney disease, hypertension, cardiovascular disease and diabetes mellitus being more commonly observed in those with severe SARS-CoV-2 infection along with the knowledge that SARS-CoV-2 enters host cells through the angiotensin-converting enzyme 2 (ACE2) receptor on the surface of pulmonary type 2 alveolar cells. The Chinese data (n = 175) reported the presence of hypokalaemia in 62% of patients with SARS-CoV-2 infection and 22% of all patients had severe hypokalaemia (serum potassium <3.0 mmol/L). Many (28%) had concomitant metabolic alkalosis and of the 20 patients who had a spot urine potassium/creatinine ratio, their degree of urine potassium wasting was significantly higher than those with normokalaemia and that this correlated with the degree of infection severity. It was also noted in two patients that their renal potassium wasting persisted until complete clinical recovery from the virus which was the case with our patient. Hypokalaemia was also documented in 41.3% of a cohort of SARS-CoV infection during the 2003 epidemic but it should be noted that the largest SARS-CoV-2 case series to date (n = 1099) did not find a major difference in serum potassium between those with mild and severe disease. 

Research into the renin-angiotensin-aldosterone system has shown that genetic polymorphisms in the ACE2 gene are associated with a difference in blood pressure response to sodium and potassium intake ^4^. One study reported that ACE2 SNPs are associated with a blood pressure reduction in response to potassium intake during a period of high salt intake where blood pressure would typically be expected to rise and that ‘potassium sensitivity’ of blood pressure may exist ^5^. Further research using the 100 000 genomes project has suggested that ACE2 polymorphisms could explain the higher risk of severe disease and death amongst males and those of African and East Asian origin in SARS-CoV-2 infection. The Chinese SARS-CoV-2 study could reflect the literature which suggests subjects with ‘severe disease’ may represent a sub-group with ACE2 polymorphisms ^6^. It is already known that rs182366225 and rs2097723 polymorphisms are more frequent in the East Asian population and increase the expression of ACE2. It may therefore be that certain ACE2 polymorphisms are a risk factor for hypokalaemia. It should also be noted that, if this is the case, then there may be a greater prevalence of hypokalaemia amongst certain ethnic populations and that the study by Chen, D. *et al.* demonstrates a higher prevalence of hypokalaemia than in other studies because of this.

Complications from hypokalaemia can be life-threatening ^7^. Hypokalaemia can result in muscle weakness and cramps, thirst and paraesthesia but when serum potassium drops below 3.0 mmol/L arrhythmias such as QTc prolongation, torsades de pointes, ventricular fibrillation and sudden cardiac death can occur ^8^. The incidence of ventricular fibrillation is five-fold higher in those with hypokalaemia in comparison to hyperkalaemia ^9^. Inadequate management of hypokalaemia in hospitalised patients has been reported in as much as 24% in one study demonstrating an at-risk group of patients and a need for therapeutic vigilance here ^10^. 

The SARS-CoV-2 pandemic gives us many reasons to be vigilant with the detection and management of hypokalaemia. First, many of the SARS-CoV-2 clinical trial drugs prolong the QTc interval which adds to the arrhythmogenic effect of hypokalaemia. Some of the trial drugs can induce hypokalaemia themselves risking severe electrolyte disturbances. Second, arrhythmia induction has more than one risk factor in this patient population, for example, hypoxia-mediated, cytokine-storm-syndrome and direct viral cardiac myocyte damage which all make these patients vulnerable to cardiac complications ^11^. Third, the use of diuretics to improve oxygenation in those with acute respiratory distress syndrome also risks hypokalaemic complications. Finally, the data for return of spontaneous circulation (ROSC) during cardiac arrest in a retrospective study (n = 136) is unfortunately very poor (13.2% achieved ROSC and less than 3% were alive at 30 days post-cardiac arrest) which reiterates the importance of vigilance for electrolyte disturbance in this patient population ^12^.

The popular mechanism for hypokalaemia remains RAS system activation and it should be noted that not all patients with RAS activation are hypokalaemic, due to the renal potassium switch mechanism ^13, 14^. Early reports demonstrate good histopathological evidence of tubular injury which seem to have a multifactorial aetiology in SARS-CoV-2 patients ^15, 16^. These include acute tubular necrosis from cytokine-storm syndrome, direct virion invasion of tubular cells, peritubular congestion, drug toxicity and pigment-cast nephropathy from rhabdomyolysis. Based on these mechanisms it seems likely that tubular injury is difficult to avoid in these patients.

Current evidence for mechanisms of renal injury in SARS-CoV-2 infection is early and much of which is based on speculation, case reports and case series. It is clear that we need more studies that measure all components of the RAS pathway in SARS-CoV-2 patients to truly determine RAS involvement and more data on serum and urine electrolytes in infected patients including the trans-tubular potassium gradient (TTKG). It is easy to measure the TTKG and measurement only requires serum and urine osmolality and potassium (urine potassium/serum potassium)/(urine osmolality/ serum osmolality). Estimation is accurate providing urine is not dilute and that urine sodium concentration is >25mmol/l so that distal sodium delivery is not a limiting factor ^17^. A urine potassium: creatinine ratio can also be used and is preferred over a spot urine potassium or 24-hour urinary potassium collection as, because creatinine is theoretically secreted in a constant state (in the absence of an GFR drop), a urine potassium: creatinine ratio corrects for urine volume variations. The TTKG is beneficial as it does not overestimate the gradient for collecting duct potassium secretion, however it is not without flaws which are discussed elsewhere ^18^.

## Conclusion

Here we summarise a case of severe SARS-CoV-2 infection with evidence of significant renal potassium wasting resulting in hypokalaemia. Complications from hypokalaemia can be life-threatening, especially during critical illness, thus we advocate checking the trans-tubular potassium gradient in patients with moderate-severe infection. This should help identify those at risk of hypokalaemia so appropriate monitoring and electrolyte replacement can be promptly instituted to prevent this potentially fatal complication.

## Consent

Written informed consent for publication of their clinical details was obtained from the patient.

## Data availability

### Underlying data

All data underlying the results are available as part of the article and no additional source data are required.
