# Supplementary material for: Case Report: Renal potassium wasting in SARS-CoV-2 infection
Source: F1000Res. 2020 Nov 13;9:659. Originally published 2020 Jun 30. [Version 2] doi: 10.12688/f1000research.24621.2 (PMC7702164; doi:10.12688/f1000research.24621.2)
Supplement: Peer review history

# Peer review: Reviewer response for version 2

**DOI:** 10.5256/f1000research.30666.r74801

**Published:** 2020-11-26

**Authors:** Ming-Hsien Tsai

**Affiliations:** 1 Division of Nephrology, Department of Internal Medicine, Shin-Kong Wu Ho-Su Memorial Hospital, Fu-Jen Catholic University School of Medicine, Taipei, Taiwan

I am satisfied with this revision.

Are enough details provided of any physical examination and diagnostic tests, treatment given and outcomes?

Partly

Is the case presented with sufficient detail to be useful for other practitioners?

Partly

Is sufficient discussion included of the importance of the findings and their relevance to future understanding of disease processes, diagnosis or treatment?

Partly

Is the background of the case’s history and progression described in sufficient detail?

Yes

Reviewer Expertise:

nephrology

I confirm that I have read this submission and believe that I have an appropriate level of expertise to confirm that it is of an acceptable scientific standard.

# Peer review: Reviewer response for version 1

**DOI:** 10.5256/f1000research.27159.r71137

**Published:** 2020-10-07

**Authors:** Ming-Hsien Tsai

**Affiliations:** 1 Division of Nephrology, Department of Internal Medicine, Shin-Kong Wu Ho-Su Memorial Hospital, Fu-Jen Catholic University School of Medicine, Taipei, Taiwan

The authors reported a case with hypokalemia may relate to SARS-Cov-2 infection. This case is well written but still, some concerns need to be clarified.

****

**Major:**

1. The timeline in this case is important for a comprehensive understanding. I suggest that the authors can modify Table 1 to show the baseline data and consequent laboratory change.
2. Did the authors have the result of urine nitrogen, creatinine, sodium, potassium, calcium, Mg, phosphate, and osmolarity levels? If yes, please provide.

**Minor:**

1. The dosed of medications that she used in OPD should be provided in the part of the case report.
2. The author should focus on this case and clearly tell us that what is the most possible etiology of renal wasting in this case.  
3. The urine K/Cr ratio can offer a more quick and reliable interpretation of the hypokalemia. I suggest the author can discuss this.

Are enough details provided of any physical examination and diagnostic tests, treatment given and outcomes?

Partly

Is the case presented with sufficient detail to be useful for other practitioners?

Partly

Is sufficient discussion included of the importance of the findings and their relevance to future understanding of disease processes, diagnosis or treatment?

Partly

Is the background of the case’s history and progression described in sufficient detail?

Yes

Reviewer Expertise:

nephrology

I confirm that I have read this submission and believe that I have an appropriate level of expertise to confirm that it is of an acceptable scientific standard, however I have significant reservations, as outlined above.

# Peer review: Reviewer response for version 1

**DOI:** 10.5256/f1000research.27159.r68472

**Published:** 2020-08-17

**Authors:** Shabbir Moochhala

**Affiliations:** 1 UCL Centre for Nephrology, Royal Free Campus and Hospital, University College London Medical School, London, UK

The authors present a well-observed, useful and much needed case and comment on an important renal complication of SARS-CoV-2 infection in a co-morbid patient.

 My main concern is that the possibility of this being a primary metabolic alkalosis (with secondary hypokalaemia) has not been adequately refuted. The blood gas data show a metabolic alkalosis with respiratory compensation, and simultaneously a relatively modest hypokalaemia for this degree of alkalosis. Contraction alkalosis (due to pyrexia causing volume depletion/hypotension) or hypercapnoeic metabolic alkalosis (hypoxic on admission with COPD) might be explanations for this ^1^. Results for chloride and CK, if available, may be helpful. 

 I agree that the results do not support aldosterone-mediated hypokalaemia. 

 In fig 1, Hartmann's and sando K on day 3 should be mentioned together for clarity. 

 I am not clear about when TTKG was measured. Presumably this was before potassium replacement on day 3 to avoid confounding, but this should be mentioned.

Are enough details provided of any physical examination and diagnostic tests, treatment given and outcomes?

Yes

Is the case presented with sufficient detail to be useful for other practitioners?

Yes

Is sufficient discussion included of the importance of the findings and their relevance to future understanding of disease processes, diagnosis or treatment?

Yes

Is the background of the case’s history and progression described in sufficient detail?

Yes

Reviewer Expertise:

Clinical renal disease, renal tubular disease

I confirm that I have read this submission and believe that I have an appropriate level of expertise to confirm that it is of an acceptable scientific standard.

## References

[1] : Hyperpyrexia and metabolic alkalosis in a COVID‐19 patient. *Journal of Medical Virology*.2020; 10.1002/jmv.26276 10.1002/jmv.26276 PMC736133532639623
